# Influenza pandemic preparedness: motivation for protection among small and medium businesses in Australia

**DOI:** 10.1186/1471-2458-7-157

**Published:** 2007-07-17

**Authors:** Rochelle E Watkins, Feonagh C Cooke, Robert J Donovan, C Raina MacIntyre, Ralf Itzwerth, Aileen J Plant

**Affiliations:** 1 Australian Biosecurity Cooperative Research Centre, Curtin University of Technology, Perth, Australia; 2 Social Marketing Research Unit, School of Marketing, Curtin University of Technology, Perth, Australia; 3 Discipline of Paediatrics and Child Health, Faculty of Medicine, University of Sydney, Sydney, Australia; 4 National Centre for Immunisation Research and Surveillance of Vaccine Preventable Diseases, Children's Hospital at Westmead, Sydney, Australia; 5 Deceased

## Abstract

**Background:**

Community-wide preparedness for pandemic influenza is an issue that has featured prominently in the recent news media, and is currently a priority for health authorities in many countries. The small and medium business sector is a major provider of private sector employment in Australia, yet we have little information about the preparedness of this sector for pandemic influenza. This study aimed to investigate the association between individual perceptions and preparedness for pandemic influenza among small and medium business owners and managers.

**Methods:**

Semi-structured face-to-face interviews were conducted with 201 small and medium business owners or managers in New South Wales and Western Australia. Eligible small or medium businesses were defined as those that had less than 200 employees. Binomial logistic regression analysis was used to identify the predictors of having considered the impact of, having a plan for, and needing help to prepare for pandemic influenza.

**Results:**

Approximately 6 per cent of participants reported that their business had a plan for pandemic influenza, 39 per cent reported that they had not thought at all about the impact of pandemic influenza on their business, and over 60 per cent stated that they required help to prepare for a pandemic. Beliefs about the severity of pandemic influenza and the ability to respond were significant independent predictors of having a plan for pandemic influenza, and the perception of the risk of pandemic influenza was the most important predictor of both having considered the impact of, and needing help to prepare for a pandemic.

**Conclusion:**

Our findings suggest that small and medium businesses in Australia are not currently well prepared for pandemic influenza. We found that beliefs about the risk, severity, and the ability to respond effectively to the threat of pandemic influenza are important predictors of preparedness. Campaigns targeting small and medium businesses should emphasise the severity of the consequences to their businesses if a pandemic were to occur, and, at the same time, reassure them that there are effective strategies capable of being implemented by small and medium businesses to deal with a pandemic.

## Background

Since late 2005 the risk of pandemic influenza and the need for preparedness have featured reasonably frequently in the news media in Australia, often associated with overseas reports of large outbreaks of infection among birds or small clusters of infection among humans. Strategic plans have been prepared for an outbreak of pandemic influenza associated with human avian influenza infection at national and global levels [1,2]. Many of these plans include mechanisms to facilitate and manage community-wide responses in recognition of the likelihood that pandemic response requirements will exceed the response capacity of health authorities and governments.

Preparation by the business community for an influenza pandemic is encouraged by governments, but much of the onus is on businesses to inform themselves about the threat posed by pandemic influenza and develop their own plans. As such, large corporations often have detailed plans, but less is known about pandemic preparedness in small and medium sized businesses. Small and medium businesses are a major employer in Australia, accounting for approximately half of all private sector employees [3]. Small businesses, which include businesses with less than 20 employees, were alone estimated to employ almost 3.6 million people in Australia in 2001 [3]. Pandemic influenza is likely to have a major impact on businesses, yet little is known about the needs and preparedness of small and medium sized businesses.

Government and health authorities in Australia and overseas have recommended that businesses, community organisations and individuals implement a range of strategies to prepare for pandemic influenza, and an increasing number of resources are being produced to provide guidance on pandemic preparedness and business continuity planning [4-7]. A resource specific to pandemic preparedness planning among small businesses in Australia is also available [8].

Pandemic planning resources generally describe the nature of the anticipated threat, highlight the role of government and health authorities, outline essential business continuity planning and response requirements, and describe specific measures that may be implemented to limit or prevent disease spread. Specific strategies recommended to limit disease spread within the workplace include promoting improved hygiene and infection control practices, using social distancing measures and flexible work arrangements to minimise contact between individuals within the workplace and the community, using personal protective equipment, restricting workplace entry and isolating individuals who may be infectious [4].

A greater understanding of the factors associated with planning for pandemic influenza among small and medium businesses is required to inform communication strategies that promote improved preparedness for a pandemic. Protection motivation theory [9] is a commonly used framework for fear-appeal research [10]. Protection motivation theory conceptualises an individual's acceptance of advice on how to protect themselves from a health threat as primarily a function of four specific beliefs: the perceived severity or seriousness of the threat and the likelihood of the threat occurring (which together constitute 'threat appraisal'); and the perceived effectiveness of actions to avoid the threat and the individual's perceived self-capacity to implement those actions (which together constitute 'coping appraisal'). If a sufficient level of threat is perceived to exist, and coping appraisal is high, then the individual will take appropriate action. However, where a threat appraisal is high but coping appraisal is low, the individual is unlikely to take appropriate action. Protection motivation theory suggests that campaigns using threats must include information about how to avert the threat, and ensure that members of the target audience have the skills and resources necessary to adopt the recommended actions.

Investigations of the effectiveness of health threat communications are supportive of the protection motivation theory framework [11], finding that communication effectiveness is associated with the extent to which the communications present real but controllable threats [12]. The health belief model [13,14] also conceptualises behaviour as dependent upon individual perceptions including the perceived likelihood and severity of the potential health threat, and the perceived effectiveness of responses to the threat. Similarly, research has supported the importance of health belief model constructs in behaviour change [15], particularly where illness avoidance and perceived threat are of central importance [16,17].

Among health behaviour theories that identify similar constructs as important determinants of health behaviour, current research provides no clear indication of the superiority of any single approach [17]. Guided by the concepts considered to be of importance in these health behaviour theories, and the protection motivation theory framework in particular, we aimed to investigate the association between selected beliefs and preparedness for pandemic influenza among small and medium business owners and managers.

## Methods

### Study design and sample

Between May and July 2006, structured face-to-face interviews were conducted with either the owners or managers of 201 small or medium businesses in Australia. Eligible businesses were defined as businesses which have less than 200 employees [3]. Participating businesses were recruited from New South Wales (101) and Western Australia (100), with approximately 70 per cent of the sample in each state being selected from businesses located in the capital cities (n = 140), 20 per cent being recruited from large satellite cities (n = 41), and the remaining 10 per cent from rural centres (n = 20).

Participating businesses in Western Australia were randomly sampled from a membership database of businesses obtained from the Local Chambers of Commerce and Industry. In New South Wales businesses were randomly sampled from a purchased list of 1500 businesses stratified by industry type. In both instances the lists of businesses were checked to ensure that the sampling frames included only businesses which operated in the eligible study areas prior to recruitment. A flow chart was used to guide the recruitment of interviewees in both states to ensure recruitment processes were standardised, including gaining confirmation that the business had less than 200 employees, ensuring that a minimum of three attempts were made to establish contact with each business to be recruited, and ensuring that an appropriate person was interviewed.

Face to face interviews with business owners or senior managers were administered by trained interviewers from a market research firm in New South Wales, and by trained interviewers contracted by the Local Chambers of Commerce and Industry in Western Australia. Prior to the study interview verbal consent to participate was obtained following the provision of, and discussion of, a study information sheet. Study procedures were approved by the Human Research Ethics Committee of Curtin University of Technology.

### Measures

Focus group discussions with business owners and managers in Perth and Sydney were used to inform and develop the structured interview schedule. The interview schedule was pre-tested among a small sample of business owners to ensure the questions were acceptable, understandable, unambiguous, and that open ended questions elicited the expected type of response.

Basic characteristics of the participating businesses assessed included the job classification of the interviewee (owner, chief executive officer/managing director, senior manager), main business location (capital city, satellite city, rural centre), industry type, business size (number of employees), average number of customers per day, and the educational level of employees (proportion of employees who attended university). The response categories for industry type were pre-coded based on the divisions in the 1993 Australian and New Zealand Standard Industrial Classification [18]. For analysis purposes, businesses operating in the primary and secondary industry sectors (i.e., businesses engaged in production and manufacturing) were aggregated; and classifications for tertiary industry businesses (i.e., businesses operating within the service sector) were aggregated according to the following three industry type categories: property and business services; retail trade; and other tertiary.

The following beliefs about pandemic influenza were each assessed by a single question: the perceived severity of the threat; the risk of the threat; and the ability to respond effectively to the threat. The general belief about the severity of the threat was operationalised as the perceived proportion of people that could become sick if pandemic influenza were to affect the local community. The perceived risk of the threat was operationalised as the likelihood that pandemic influenza would become a significant health issue in Australia in the near future, and assessed on a four point scale (very unlikely, unlikely, likely, very likely). An additional business-specific indicator of risk, the perceived level of risk that pandemic influenza poses to the interviewee's business, was also rated on a four-point scale (no risk, some risk, moderate risk, high risk). A dichotomous indicator of coping appraisal was derived from the open-ended question: "Can you think of any steps you can take to protect your business from pandemic influenza?" Responses were independently reviewed by two coders, and participants who were unable to identify any potentially useful steps that could be taken to protect their business or limit disease spread were classified as having low coping appraisal.

A small proportion of participants nominated the responses 'don't know' and 'no idea' to represent their beliefs about the risk and severity of pandemic influenza (Table 1). When dichotomous indicators of risk and severity were used in the analysis, these responses were aggregated with the other low risk or low severity responses for analysis purposes on the basis that these responses indicated an absence of perceptions of high risk or high severity. This coding did not significantly affect the findings of the analysis.

**Table 1:** Characteristics of participating businesses, beliefs about pandemic influenza and dependent variables according to state

**Characteristic (n = 201)**	**WA** n (%)	**NSW** n (%)	p*	**Total** n (%)
Interviewee			0.000	
Owner	62 (62.0)	46 (45.5)		108 (53.7)
Chief executive officer/managing director	12 (12.0)	37 (36.6)		49 (24.4)
Senior manager	26 (26.0)	18 (17.8)		44 (21.9)
Industry type			0.003	
Primary or secondary	9 (9.0)	11 (10.9)		20 (10.0)
Tertiary: property and business services	18 (18.0)	27 (26.7)		45 (22.4)
Tertiary: retail trade	35 (35.0)	13 (12.9)		48 (23.9)
Tertiary: other	38 (38.0)	50 (49.5)		88 (43.8)
Size			0.10	
Small (<20 employees)	82 (82.0)	73 (72.3)		155 (77.1)
Medium (20–200 employees)	18 (18.0)	28 (27.7)		46 (22.9)
Severity of pandemic influenza			0.08	
Don't know	2 (2.0)	8 (7.9)		10 (5.0)
Low (<30% sick)	44(44.0)	34 (33.7)		78 (38.8)
High (30% or more sick)	54 (54.0)	59 (58.4)		113 (56.2)
Risk of pandemic influenza			0.23	
Don't know	6 (6.0)	8 (7.9)		14 (7.0)
Very unlikely	8 (8.0)	14 (13.9)		22 (10.9)
Unlikely	49 (49.0)	42 (41.6)		91 (45.2)
Likely	33 (33.0)	27 (26.7)		60 (29.9)
Very likely	4 (4.0)	10 (9.9)		14 (7.0)
Risk to business			0.36	
No idea	8 (8.0)	4 (4.0)		12 (6.0)
No risk	15 (15.0)	8 (7.9)		23 (11.4)
Some risk	29 (29.0)	33 (32.7)		62 (30.8)
Moderate risk	23 (23.0)	26 (25.7)		49 (24.4)
High risk	25 (25.0)	30 (29.7)		55 (27.4)
Thought about pandemic influenza			0.46	
Not at all	40 (40.0)	39 (38.6)		79 (39.3)
A little	44 (44.0)	39 (38.6)		83 (41.3)
A lot	16 (16.0)	23 (22.8)		39 (19.4)
Response efficacy			0.003	
Low	51 (51.0)	31 (30.7)		82 (40.8)
High	49 (49.0)	70 (69.3)		119 (59.2)
Need help to prepare			<0.001	
Yes	45 (45.0)	76 (75.2)		121 (60.2)
Unsure	20 (20.0)	6 (5.9)		26 (12.9)
No	35 (35.0)	19 (18.8)		54 (26.9)
Pandemic influenza plan			0.047	
Yes	3 (3.0)	10 (9.9)		13 (6.5)
No/Unsure	97 (97.0)	91 (90.1)		188 (93.5)

* Chi square test of association

Western Australia (WA)

New South Wales (NSW)

Three dependent variables in the analysis provide different indicators of engagement in adaptive processes associated with the threat of pandemic influenza. Participants were asked "Before being contacted about this study, how much have you thought about the impact of pandemic influenza on your business?" (not at all, a little, a lot). The need for help with planning for pandemic influenza, which can be considered an indicator of an adaptive response to the threat of pandemic influenza, was assessed using the following open-ended question: "Is there anything you need to help you prepare for pandemic influenza?" Responses were dichotomised into a variable which indicated whether help was or was not required. Lastly, the presence of a plan for pandemic influenza was assessed by the single question "Has your business made any specific plans should pandemic influenza arise?" (yes, no, unsure).

### Analysis

The chi-square test of independence was used to test for associations between categorical study variables, and the independent samples t-test was used to test for differences between groups on continuous variables. Phi, which is a measure based on the chi-square test of association, is used to assess the strength of association between two dichotomous variables, and indicates the amount of total variance explained by the association between the variables.

Binomial logistic regression analysis was used to identify the significant independent predictors of the health behaviour theory-based belief variables and the three main dependent variables: having considered the impact of, having a plan for, and needing help to prepare for pandemic influenza.

Dependent variables were dichotomised for analysis due to skewed distributions and the small sample size. Initial model development included entry of variables into a forward stepwise model, with the probability criterion for entry set at 0.05 and exit at 0.10. The final models were developed manually to allow exploration of alternative model forms. A main effects model was initially determined. Effect modification was also explored, and the inclusion of interactions was determined by the significance of the change in log likelihood of the model. Crude odds ratios (COR), adjusted odds ratios (AOR) adjusted for the other variables in each model, and 95% confidence intervals (95%CI) are used to summarise the magnitude of association found between variables. All analyses were performed using SPSS version 13.0 (SPSS Inc., 2004) and the significance level was set at p ≤ 0.05.

## Results

### Sample description

In total, 832 eligible businesses were contacted and 201 interviews were completed, producing an overall response rate of 24 per cent. The response rate of 15 per cent (101/660) for New South Wales (NSW) was considerably lower than the 58 per cent (100/172) achieved for Western Australia (WA), but consistent with the different sampling methods used. There was no significant difference between participating and non-participating businesses in WA according to business size (p = 0.3) or industry type (p = 0.09). Similar data on the characteristics of non-participating businesses in NSW were not available for analysis. Non-participation was most frequently associated with the business owner or manager being either too busy or unavailable during the interview period, explaining 83 per cent and 85 per cent of refusals in the WA and NSW samples respectively.

The characteristics of participating businesses are summarized in Tables 1 and 2 by state. Most participating businesses had less than 20 employees and more than half of the individuals interviewed were business owners. Business owners were significantly more likely to be interviewed in WA than NSW (Table 1). Most of the participating businesses operated within the tertiary or service sector. The representation of businesses from different industry types was significantly different by state, with the WA sample having a higher proportion of retailers (Table 1) and reporting a significantly lower proportion of university educated staff compared with the NSW sample (Table 2).

**Table 2:** Characteristics of participating businesses and beliefs about pandemic influenza according to state

**Variable**	**WA**	**NSW**	p*	**Total**
	mean (SD)	mean (SD)		mean (SD)
Number of employees (n = 201)	14.6 (27.5)	23.1 (36.5)	0.07	18.9 (32.6)
Number of customers (n = 201)	47.7 (88.8)	75.5 (345.5)	0.44	61.7 (252.5)
Per cent of staff university educated (n = 200)	25.7 (33.9)	44.4 (32.9)	<0.001	35.2 (34.6)
Severity of pandemic influenza (n = 191)				
Per cent sick	32.6 (20.4)	36.6 (24.3)	0.22	34.6 (22.4)

*Independent samples t-test

Western Australia (WA)

New South Wales (NSW)

Standard deviation (SD)

Approximately 40 per cent of participants believed that pandemic influenza was likely or very likely to become a significant health issue in Australia in the near future (Table 1), and, on average, participants believed 35 per cent of people in affected communities would become sick (Table 2). Around 40 per cent of participants reported that they had not spent any time thinking about the impact of pandemic influenza on their business, and over 40 per cent could not identify any steps that they could take to protect their business (Table 1). Only 6 per cent of participants reported having a pandemic influenza plan for their business (3 per cent were unsure), and over 60 per cent of participants reported needing help to prepare for pandemic influenza (Table 1).

Beliefs about the risk and severity of pandemic influenza and the amount of time spent considering the impact of pandemic influenza on the business did not differ significantly between states. Beliefs about being able to respond to the threat and perceptions about the need for help did differ between states, with businesses in WA having a significantly lower level of response efficacy and being less likely to need help to prepare than businesses in NSW (Table 1). Businesses in NSW were also significantly more likely to have a plan for pandemic influenza than businesses in WA (Table 1); however, the difference in response rate for the two states renders the generalisability of such differences as tenuous.

### Business characteristics and beliefs

Beliefs about the perceived severity of pandemic influenza and the perceived risk of pandemic influenza to the business were not significantly associated with any business characteristics. Business characteristics which were significant predictors of beliefs about the perceived risk of pandemic influenza and coping appraisal are summarized in Table 3. The perceived risk of apandemic in Australia was significantly associated with the role of the person interviewed, with non-owners being about twice as likely to consider pandemic influenza as a likely or very likely risk than business owners. Both state and the role of the individual interviewed were significantly associated with response efficacy, with businesses in NSW and non-owners being about twice as likely to be able to identify actions which could protect their business in the event of a pandemic than businesses in WA and owners. Each of the significant predictors of beliefs identified only explained a small proportion (less than 5 per cent) of the overall variance associated with the belief variables.

**Table 3:** Significant independent predictors of belief variables

**Belief predictors (n = 201)**	**Adjusted OR (95% CI)**	Wald statistic*	**p**
**Risk of pandemic influenza (Likely or Very likely)**			
Interviewee (ref: Owner)			
Other	2.0 (1.1 – 3.5)	5.12	0.02
**Response efficacy (High)**			
State (ref: WA)			
NSW	2.2 (1.2 – 3.9)	6.57	0.01
Interviewee (ref: Owner)			
Other	1.9 (1.1 – 3.5)	4.65	0.03

*The Wald statistic indicates whether the independent variable is significantly associated with the dependent variable

Odds ratio (OR)

### Predictors of dependent variables

Bivariate associations between beliefs and the dependent variables (Table 4) indicate that almost all beliefs and dependent variables assessed were significantly associated. The high correlation between the general belief about the risk of pandemic influenza and the specific belief about the risk of pandemic influenza to the business, which explained over 37 per cent of the total variance in responses (equivalent to a Pearson correlation coefficient of approximately 0.6), was among the strongest associations found. There was no significant association between having a plan and the need for help, and coping appraisal was independent of perceptions of severity.

**Table 4:** Bivariate associations between selected belief variables and dependent variables

**Variable (abbreviated name)**	Phi* (p)					
	
	**Severity**	**Risk a**	**Risk b**	**Thought**	**Efficacy**	**Help**
Severity of pandemic influenza (Severity)	-	-	-	-	-	-
(30% or more sick)						
Risk of pandemic influenza (Risk a)	0.28	-	-	-	-	-
(Likely or very likely)	(<0.001)					
Risk to business (Risk b)	0.39	0.37	-	-	-	-
(Moderate or high risk)	(<0.001)	(<0.001)				
Thought about pandemic influenza (Thought)	0.21	0.30	0.17	-	-	-
(A lot)	(0.004)	(<0.001)	(0.02)			
Response efficacy (Efficacy)	0.10	0.15	0.17	0.15	-	-
(High)	(0.14)	(0.03)	(0.02)	(0.03)		
Need help to prepare (Help)	0.18	0.18	0.27	0.25	0.19	-
(Yes)	(0.009)	(0.01)	(<0.001)	(0.001)	(0.006)	
Pandemic influenza plan	0.19	0.18	0.17	0.28	0.18	0.05
(Yes)	(0.007)	(0.01)	(0.01)	(<0.001)	(0.01)	(0.49)

* Phi indicates the proportion of the total variance explained

Logistic regression models were used to determine the significant independent predictors of having considered the impact of, having a plan for, and needing help to prepare for a pandemic. All models were tested for interaction terms and no significant effect modification was found. The significant independent predictors of dependent variables, based on the inclusion of both belief variables and business characteristics, are summarised in Table 5.

**Table 5:** Significant independent predictors of dependent variables

**Dependent variable predictors (n = 201)**	**Adjusted OR (95% CI)**	**Wald** statistic*	**p**
**Thought about pandemic influenza (A lot)**	**Model R^2 ^= 0.18**		
Risk of pandemic influenza (ref: Unlikely/very unlikely/don't know)			
Likely or very likely	5.1 (2.4 – 10.8)	17.39	<0.001
Location (reference: Non-capital city)			
Capital city	3.2 (1.2 – 8.4)	5.51	0.02
**Need help (Yes)**	**Model R**^2^** = 0.27**		
Risk to business (ref: Low risk/no risk/no idea)			
Moderate or High	3.3 (1.7 – 6.3)	13.3	<0.001
State (reference: WA)			
NSW	3.3 (1.7 – 6.4)	12.7	<0.001
Industry type (ref: Tertiary: other)		11.3	0.01
Primary or secondary	0.8 (0.3 – 2.6)	0.13	0.72
Tertiary: property and business services	0.4 (0.2 – 0.9)	5.04	0.03
Tertiary: retail trade	0.3 (0.1 – 0.7)	8.94	0.003
**Pandemic influenza plan (Yes)**	**Model R^2 ^= 0.20**		
Severity of pandemic influenza (ref: < 30% sick/no idea)			
30% or more sick	9.3 (1.2 – 73.9)	4.47	0.04
Response efficacy (reference: low)			
High	8.1 (1.0 – 64.4)	3.92	0.048

*The Wald statistic indicates whether the independent variable is significantly associated with the dependent variable

Odds ratio (OR)

Thinking a lot (versus a little or not at all) about the impact of pandemic influenza on the business was most strongly associated with the perceived risk of pandemic influenza, with participants who perceived a pandemic as likely or very likely to be a health issue in Australia in the near future being approximately 5 times more likely to have reported thinking a lot about the impact of a pandemic on their business. Businesses that were located in the capital city were about three times more likely to have spent a lot of time thinking about the impact of a pandemic compared with businesses in satellite city or rural locations. These same factors were also significant predictors of having considered the impact of a pandemic on the business when this variable was dichotomised as thought at all (a little or a lot) versus not at all.

The perceived need for help was most strongly associated with the perceived risk of pandemic influenza to the business, with participants who perceived the risk of a pandemic to the business as moderate or high being approximately 3 times more likely to report needing help to prepare. State was a significant independent predictor of the perceived need for help, with businesses in NSW more likely to report needing help than those in WA. The perceived need for help was also significantly associated with industry type, with businesses in the property and business services and retail trade sectors being significantly less likely to need help than other service sector businesses. Industry type was not significantly associated with perceptions about the risk or severity of a pandemic, but was significantly associated with coping appraisal (χ^2 ^= 8.4, p = 0.04), with 58 per cent of retailers unable to think of steps to protect their business as opposed to 35 per cent of other service sector businesses and 30 per cent of production and manufacturing businesses.

The presence of a specific plan for pandemic influenza was significantly and independently associated with both perceived severity of a pandemic and coping appraisal. Participants who believed that 30 per cent or more of the local community would become sick were over 9 times more likely to have a plan, and participants who were able to identify steps that could be taken to protect their business were over 8 times more likely to have a plan for pandemic influenza.

## Discussion

There is a lack of empirical data to inform public health response strategies for pandemic influenza. To our knowledge this study provides the first systematically collected information on preparedness among small and medium businesses in Australia, and is among only a few studies in the field worldwide. We found that only a small proportion of businesses studied had thought a lot about how pandemic influenza may impact on their business, that few had made any specific plans to protect their staff or their business in the event of pandemic influenza, and that over 60 per cent state they need help to prepare for pandemic influenza. These findings suggest that additional strategies are required to promote increased awareness of the threat of pandemic influenza in the community, to promote the resources available to assist with preparedness, and to facilitate engagement in preparedness planning.

Behaviour change is a process, and time is required to initiate and establish new behaviours. According to the protection motivation theory, coping appraisal responses which lead to the establishment of protection motivation occur after the threat-appraisal process, as a threat needs to be identified before coping options can be evaluated [11]. As such, and as has already been highlighted by others, occasional media reports are insufficient to adequately inform individuals about pandemic influenza, and interventions are required before a pandemic occurs to improve public awareness, build mutual trust, promote effective coping responses and assist in the successful implementation of plans when they are required [19].

National influenza plans require collective community-wide efforts for an effective response to pandemic influenza. However, they lack information relating to strategies to enable the effective dissemination of this information beyond the availability of these plans on websites [19]. Given that the strategy for response to pandemic influenza in Australia is based on containment and reducing transmission of the virus [2], and that key response strategies such as isolation, social distancing, and improved personal hygiene which have been supported by mathematical modelling studies [20] depend on community-wide behaviour modification, additional strategies are required to enable an effective shared response.

Our findings suggest that the beliefs of small and medium business owners and managers are likely to have important consequences for preparedness. Beliefs about the risk of and severity of pandemic influenza were the most important independent predictors of having thought about, and having a plan for pandemic influenza respectively. The perceived risk of pandemic influenza to the business was also the most important predictor of needing help to prepare. These findings are consistent with the relationships proposed by prominent theories of health behaviour, including the protection motivation theory [9,11], and suggest that these theories provide a useful model for understanding preparedness behaviours among small and medium businesses in Australia and elsewhere.

Protection motivation theory and health belief model concepts have been found to be valuable for understanding and promoting a variety of health-related behaviours [10,12,21,22], including the performance of protective behaviours during the outbreak of the severe acute respiratory syndrome in Hong Kong [16]. The importance of perceptions about risk and severity in understanding preparedness behaviour suggests that health behaviour theories provide a useful framework for the design of communication strategies that aim to promote preparedness for pandemic influenza among the business community. Based on the temporal relations identified in these theoretical frameworks, our results suggest that communications containing information about risk and severity are likely to promote both threat appraisal and coping appraisal processes, and can motivate protective behaviours given a perceived ability to implement recommended actions. Promotion of the ability to respond effectively to the threat of pandemic influenza appears to be an important factor associated with protective responses to the threat of pandemic influenza. This finding is consistent with research findings based on other health threats which indicates that low levels of self efficacy and response efficacy provide a barrier to action [11,12]. The high proportion of participants reporting needing help with preparation indicates that self efficacy may be an important factor limiting planning for pandemic influenza, which is consistent with the findings of recent research in Europe and Asia [23].

Individual business characteristics were relatively unimportant among the predictors of having thought about or planned for pandemic influenza. Apart from beliefs about risk, the only other significant predictor of having considered the impact of pandemic influenza on the business was whether the business operated within or outside a capital city. It is possible that this association reflects a factor which can modify the perceived threat of pandemic influenza based on understandings about population density and the probability of exposure to infection.

In contrast, individual business characteristics were more important predictors of needing help to prepare, with industry type and state being significant predictors in addition to beliefs about risk. Retail traders and businesses that provide property and business services were less likely to report the need for help. Differences in the need for help by industry type, given the significant association between industry type and coping appraisal, suggests that some businesses may have difficulty identifying effective protection strategies that are appropriate for specific high-risk business environments, such as retail outlets. This finding highlights the importance of providing support to identify effective response strategies and overcome response difficulties within all business environments. Furthermore, our finding that the need for help was not significantly related to whether a plan for pandemic influenza exists appears to highlight the difficulties associated with planning for pandemic influenza, even among those businesses that have already made specific plans for pandemic influenza.

Our finding of a difference in the need for help by state is likely to be associated with the different sampling and recruitment processes used in the two study locations. In WA the Local Chambers of Commerce was directly contracted to supply the business contact details and conduct the interviews. Thus, the existing relationship with the businesses sampled is likely to explain the higher response rate in WA, why a higher proportion of owners were interviewed, and provide a sample which may be less biased in terms of either having a specific interest in pandemic influenza or time or resource pressures than the NSW sample. Selection bias associated with the different recruitment strategies may explain why participants from NSW were more likely to have a plan, were more likely to need help and reported lower response efficacy. Alternatively, these findings may be due to real differences in beliefs and behaviour between states, which may for example be associated with differences in media exposure or other local influences. Regardless of the cause, these differences did not significantly influence the associations found between beliefs and preparedness.

Due to the cross-sectional study design we are limited in the type of conclusions that we can draw about causality based on the associations observed. For example, having prepared a pandemic influenza plan is likely to result in improved levels of coping appraisal. However, experimental research [24] has provided support for the impact of beliefs on protection motivation and current behaviour. The associations found in this study explained a low proportion of variance in preparedness behaviour, although the magnitude of the associations found is similar to those reported for protection motivation theory concepts and other health-related behaviours [24,25]. Several factors could have contributed to the low explanatory power in the present study, including the assessment of a limited number of theory-based belief constructs, the use of single-item and thus limited operationalisations of the key belief and outcome variables which have unknown reliability, and the use of dichotomous indicators due to the small sample size. Also we did not assess behavioural intentions. Further work is required to extend the scope of this study and considered other relevant constructs including social norms and response costs.

The non-random nature of the sampling frames used to recruit study participants and the small scale of the study limits the generalisability of the study findings. It is also likely that response bias associated with the low response rate may have resulted in an overestimation of the proportion of businesses that have a plan for pandemic influenza, particularly in NSW. The use of financial or other incentives for participation is recommended in future studies to facilitate improved response rates, particularly where industry partners are not used. The findings of this study may also be limited in that self-report methods were used to assess whether the business had a pandemic influenza plan. Responses may have been biased in favour of reporting the presence of a plan or having considered the impact of pandemic influenza on the business associated with social desirability bias.

There is a shortage of data available to guide public health policy and practice in pandemic influenza planning and response [6]. Current guidance for pandemic influenza preparedness appears to have had little impact on preparedness among the small and medium business sectors in Australia. Our findings suggest that further investment by governments is required to improve both the specification of and utilisation of available planning resources, as has been highlighted previously [26]. Further work is required to underpin both the design of communication strategies to promote behavioural change, as well as the feasibility and effectiveness of strategies for disease control, which also support beliefs about being able to respond effectively to the threat of pandemic influenza.

The findings of this study should be interpreted alongside more in-depth knowledge about the beliefs of business owners and managers that underlie the protection motivation theory constructs, as has been illustrated elsewhere [27]. In this way, a greater understanding about beliefs to be reinforced or changed, and responses to specific strategies can be gained, helping to promote improved effectiveness of the communication strategies developed. Also, particularly in the small and medium business sectors that may have significant resource constraints, the presence of alternative adaptive responses to the threat of pandemic influenza require further investigation.

## Conclusion

We found that only a small proportion of small and medium sized businesses in Australia have made formal plans to guide their response in the event of pandemic influenza. Effective communication strategies and support structures to promote preparedness for pandemic influenza are essential to facilitate large-scale community involvement in response efforts. Findings from this study provide knowledge which can be used in the preparation of strategies to enable the effective delivery of information on preparedness for businesses. Our results indicate that to motivate improved planning among the small and medium business sector, campaigns targeting small and medium businesses should emphasise the severity of the consequences to their businesses if a pandemic were to occur, and, at the same time, reassure them that there are effective strategies capable of being implemented by small and medium businesses to deal with a pandemic.

## Competing interests

The author(s) declare that they have no competing interests.

## Authors' contributions

AJP, REW and CRM conceived, designed and supervised the study; FCC and RI entered the data; REW and FCC analyzed the data; REW drafted the manuscript; and RJD, AJP, CRM and FCC provided feedback on the interpretation of results and editorial comments on the manuscript.

## Dedication

We wish to dedicate this paper to the memory of our colleague and much loved friend Professor Aileen Joy Plant who died suddenly on the 27th of March 2007 while on an avian influenza mission for the World Health Organization. This work would not have been possible without her leadership. Her outstanding vision for and contribution to the advancement of global public health will be greatly missed.

## Pre-publication history

The pre-publication history for this paper can be accessed here:


